# Survival of free-living *Acholeplasma* in aerated pig manure slurry revealed by ^13^C-labeled bacterial biomass probing

**DOI:** 10.3389/fmicb.2015.01206

**Published:** 2015-10-31

**Authors:** Dai Hanajima, Tomo Aoyagi, Tomoyuki Hori

**Affiliations:** ^1^Dairy Research Division, Hokkaido Agricultural Research Center, National Agricultural and Food Research OrganizationSapporo, Japan; ^2^Environmental Management Research Institute, National Institute of Advanced Industrial Science and TechnologyTsukuba, Japan

**Keywords:** stable isotope probing, dead bacterial biomass, assimilation, heterotroph, aerated slurry, *Acholeplasma*

## Abstract

Many studies have been performed on microbial community succession and/or predominant taxa during the composting process; however, the ecophysiological roles of microorganisms are not well understood because microbial community structures are highly diverse and dynamic. Bacteria are the most important contributors to the organic-waste decomposition process, while decayed bacterial cells can serve as readily digested substrates for other microbial populations. In this study, we investigated the active bacterial species responsible for the assimilation of dead bacterial cells and their components in aerated pig manure slurry by using ^13^C-labeled bacterial biomass probing. After 3 days of forced aeration, ^13^C-labeled and unlabeled dead *Escherichia coli* cell suspensions were added to the slurry. The suspensions contained ^13^C-labeled and unlabeled bacterial cell components, possibly including the cell wall and membrane, as well as intracellular materials. RNA extracted from each slurry sample 2 h after addition of *E. coli* suspension was density-resolved by isopycnic centrifugation and analyzed by terminal restriction fragment length polymorphism, followed by cloning and sequencing of bacterial 16S rRNA genes. In the heavy isotopically labeled RNA fraction, the predominant ^13^C-assimilating population was identified as belonging to the genus *Acholeplasma*, which was not detected in control heavy RNA. *Acholeplasma* spp. have limited biosynthetic capabilities and possess a wide variety of transporters, resulting in their metabolic dependence on external carbon and energy sources. The prevalence of *Acholeplasma* spp. was further confirmed in aerated pig manure slurry from four different pig farms by pyrosequencing of 16S rRNA genes; their relative abundance was ∼4.4%. Free-living *Acholeplasma* spp. had a competitive advantage for utilizing dead bacterial cells and their components more rapidly relative to other microbial populations, thus allowing the survival and prevalence of *Acholeplasma* spp. in pig manure slurry.

## Introduction

Composting is a waste treatment process by which organic material is degraded and stabilized to produce fertilizer and amend soil. Aerobic bacteria are the most important decomposers during the composting process; they obtain energy by oxidizing organic material, especially the carbon fraction. Composting is typically applied to solid waste; however, aerobic treatment is also effective for liquid waste. Animal manure slurry consisting of a mixture of feces and urine is normally stored in a manure pit until land application. During storage, malodorous compounds such as volatile fatty acids and amines, indoles, phenols, ammonia, and sulfur-containing compounds accumulate due to the anaerobic degradation of organic matter (Zhu, 2000). When the stored slurry is then directly applied to agricultural fields, it often leads to complaints from residents of surrounding areas. In addition, the application of excessive amounts of easily digestible organic matter is phytotoxic to plants. Forced aeration of manure slurry has been employed to mitigate slurry odor and stabilize its quality.

In batch solid/liquid composting, the majority of organic carbon is derived from a single initial input present within the substrate itself. Labile substrates are consumed first by copiotrophic microbial taxa, which are later replaced by more oligotrophic taxa that metabolize the remaining more recalcitrant organic carbon pools (Fierer et al., 2010). It is hypothesized that microbial populations that arise due to slurry aeration are responsible for degrading the complex mixture of organic matter such as intestinal cells, microbial cells, undigested feed, or soluble organic carbons, including odorous substances. Within several hours of initiating aeration, a distinct shift in bacterial community structure occurs, accompanied by drastic changes in slurry conditions, including physicochemical parameters such as pH, oxidation-reduction potential (ORP), and reducing organic carbon (Hanajima et al., 2009, 2011). Bacteria act as important decomposers in an aerated slurry environment, while decayed bacterial cells can serve as readily digested substrates for other microbial populations. Biomass accumulation is often highest in the earlier stages of the composting process, tapering off as succession progresses (Fierer et al., 2010). Decay of the accumulated biomass releases ammonia during the decomposition process and may trigger the regrowth of unfavorable microbial populations (e.g., pathogens).

There have been many studies on microbial community succession and/or predominant taxa during the composting process (Ishii and Takii, 2003; Danon et al., 2008; Hanajima et al., 2009, 2011). However, the ecophysiological roles of microorganisms are not well understood because microbial community structures are highly diverse and dynamic. Stable isotope probing (SIP) has been used to investigate the link between the metabolic functions and phylogenetic identities of uncultured microorganisms in natural environments (Hori et al., 2007; Lee et al., 2011). For instance, SIP of rRNA has been used to trace the incorporation of ^13^C-labeled substrates by microorganisms, which has advanced our understanding of metabolically active microbial species in a particular environment. Isotopically labeled rRNA is density-resolved by isopycnic centrifugation, and the density fraction of rRNA is analyzed by fingerprinting techniques and subsequently identified by cloning and sequencing. It was previously reported that carbon from bacterial biomass is incorporated into a soil microbial food web (Lueders et al., 2006; Murase et al., 2011); however, carbon flow mediated by microbial communities has been poorly studied in copiotrophic environments such as solid/liquid compost.

The objective of the present study was to identify active heterotrophic bacteria that are able to directly incorporate the dead *Escherichia coli* cells and their components present in aerated pig manure slurry. To achieve this objective, *E. coli* cells were cultivated with ^13^C-labeled and unlabeled glucose to prepare ^13^C-labeled and unlabeled bacterial biomass, respectively. These cells were heat-inactivated and dead cells and their components were added as substrates to aerated pig manure slurry. To clarify the fate of dead cells and their components, the incorporation of the substrate-derived ^13^C into bacterial rRNA was investigated. After identifying the microorganisms that assimilated carbon from dead bacterial cells and their components by using SIP of rRNA, their widespread distribution in several pig manure slurry was examined by pyrosequencing of 16S rRNA genes.

## Materials and Methods

### SIP with ^13^C-labeled Bacterial Biomass in Aerated Pig Manure Slurry

#### Preparation of Pig Manure Slurry

Fresh pig feces were collected from a pig fattening house at the National Institute of Livestock and Grassland Science (Tsukuba, Japan). The feces were transported to Hokkaido Agricultural Research Center (Sapporo, Japan) in a cold box, and mixed with distilled water (dry weight of feces:weight of distilled water = 1:14). The mixture was filtered through a metallic sieve (0.5-mm mesh size) to remove large suspended solids that resist biological degradation. We simulated the actual content of pig manure slurry by adding 2 g urea instead of urine to 1 l of the filtered mixture.

#### Preparation of ^13^C-labeled Bacterial Cells as a Substrate for SIP

To obtain ^13^C-labeled bacterial biomass, *E. coli* strain NBRC 3301 was cultured on mineral medium [(6.97 g l^-1^ Na_2_HPO_4_, 3.42 g l^-1^ KH_2_PO_4_, 0.2 g l^-1^ MgCl_2_⋅7H_2_O, 1.0 g l^-1^ NH_4_Cl, 0.1 g l^-1^ CaCl_2_, 0.01 g l^-1^ FeSO_4_, 0.2 mg l^-1^ ZnSO_4_, and 0.2 mg l^-1^ MnSO_4_ (pH 7.2)] (Kindler et al., 2006) containing 2 g l^-1^ ^13^C-labeled glucose (D-glucose–^13^C_6_, min. 99 atom% ^13^C; Sigma–Aldrich, St. Louis, MO, USA). Unlabeled cells for the control experiment were obtained by cultivation on the same medium except for containing unlabeled glucose in place of ^13^C-labeled glucose. Cultures were incubated on a rotary shaker at 37°C and 200 rpm. *E. coli* cultures were incubated for 16 h and cells were harvested by centrifugation (4,000 × *g*, 10 min, 4°C) and washed three times with phosphate-buffered saline (PBS; pH 7.2). The labeling of *E. coli* cells was 97.5 ^13^C atom%. ^13^C-labeled or unlabeled *E. coli* cell pellets were resuspended in 35-ml PBS and the number of viable cells in the suspension was counted by plating on LB agar (BD Biosciences, Franklin Lakes, NJ, USA). ^13^C-labeled and unlabeled *E. coli* suspensions (2.2 × 10^10^ CFU/ml) were inactivated by heating at 90°C for 30 min and stored at -20°C until use. The suspensions contained ^13^C-labeled and unlabeled bacterial cell components possibly including cell wall and membrane, as well as intracellular materials (e.g., amino acids and fatty acids).

#### Batch Treatment of Aerated Pig Manure Slurry Supplemented with ^13^C-labeled Biomass

Two batch aerobic treatments were carried out in a 5-l jar fermenter (TJM-502WS; Takasaki Scientific Instruments Corp., Saitama, Japan) containing 1.5-l manure slurry and equipped with a foam cutter. The liquid was maintained at 40°C and stirred at 350 rpm. Constant airflow at 50 ml min^-1^ l^-1^ was provided from the bottom of the jar fermenter through a ceramic stone diffuser (1.2 cm inner diameter × 5.5 cm) for 5 days. This airflow rate corresponds to the average value used by farmers in Japan (1–5 m^3^ air h^-1^ m^-3^ slurry) (Japan Livestock Industry Association, 1989). On day 3 after aeration was initiated, 30-ml suspensions of ^13^C-labeled dead *E. coli* and unlabeled dead *E. coli* (control) were added to the aerated slurry. Every 30 min, the ORP and pH of the slurry were automatically measured and recorded (Thermodac EF, Model 5020A; Eto Electrics, Tokyo, Japan). The slurry in the reactor was periodically sampled using a syringe. Samples were stored at -80°C for subsequent molecular analyses.

#### Physicochemical Analyses of Aerated Slurry

Moisture content was determined by drying samples for 24 h at 105°C. Chemical oxygen demand (COD_Cr_) was measured using the closed reflux colorimetric method in reaction tubes with prepared reagents provided by Hach Company (Loveland, CO, USA), according to the manufacturer’s instructions. Analysis of δ^13^C was carried out at the International Research Center for Agricultural Sciences (Tsukuba, Japan) using an infrared mass spectrometer (Finnigan Delta PLUS XP; Thermo Scientific, Hamburg, Germany) connected to an element analyzer (EA Flash 1112; Carlo Erba, Milan, Italy). To detect total carbon, freeze-dried slurry samples were incinerated in the element analyzer furnace and separated as pure CO_2_ gas, a small quantity of which was used to measure the ratio of ^13^CO_2_/^12^CO_2_ as different mass weights of 45/44 to obtain δ^13^C (‰).

#### RNA Extraction and Density Gradient Centrifugation

RNA was extracted from 0.5 ml of slurry samples that were obtained after 2- and 6-h incubations with ^13^C-labeled and unlabeled dead *E. coli* cells using a direct lysis protocol involving bead beating (Noll et al., 2005). Total RNA was quantified using the Ribogreen RNA quantification kit (Invitrogen, Karlsruhe, Germany) according to the manufacturer’s instructions. RNA extracts (500 ng RNA) from ^13^C-labeled and unlabeled samples were mixed with cesium trifluoroacetate (CsTFA) (Wako Pure Chemical Industries Ltd., Osaka, Japan) solution. The mixture was subjected to equilibrium density gradient centrifugation as previously described (Lueders et al., 2004). The RNA density gradients were fractionated, and their CsTFA buoyant density (BD) was determined (Lueders et al., 2004).

#### Reverse Transcription (RT)-PCR and Terminal Restriction Fragment Length Polymorphism (T-RFLP) Analysis

Each density fraction of RNA from ^13^C-labeled and unlabeled samples was subjected to RT-PCR using a PrimeScript RT-PCR kit (Takara Bio, Otsu, Japan) for T-RFLP fingerprinting. The PCR primer set 27f (5′-AGA GTT TGA TCM TGG CTC AG-3′-6-carboxyfluorescein) and 926r (5′-CCG TCA ATT CCT TTR AGT TT-3′) was used to amplify bacterial 16S rRNA genes. RT was carried out at 65°C for 5 min for annealing, followed by 42°C for 30 min and 95°C for 5 min for transcription. The thermal profile for PCR amplification consisted of 20 cycles of 94°C for 30 s, 52°C for 30 s, and 72°C for 1 min under stringent conditions, followed by final extension at 72°C for 5 min. RT-PCR products were verified by electrophoresis on a 1.5% agarose gel and were purified using the MonoFas DNA purification kit (GL Sciences, Tokyo, Japan). Two hundred nanograms of the amplicon was digested with *Msp*I (Nippon Gene, Tokyo, Japan) and then desalted by ethanol precipitation. Prior to electrophoresis, 1 μl of the digests was resuspended in 12 μl Hi-Di formamide (Applied Biosystems, Foster City, CA, USA) and 0.5 μl of GeneScan 600 LIZ Size Standard (Applied Biosystems). The mixture was denatured at 95°C for 3 min and cooled immediately on ice. Size separation of T-RFs was performed using an ABI 310 genetic analyzer (Applied Biosystems).

#### Cloning and Sequencing of the RNA Density Fractions

Selected density fractions of RNA were amplified for cloning using the primer set 27f/926r under the thermal conditions described above. RT-PCR products were ligated into the pGEM-T Easy vector (Promega, Madison, WI, USA) and the ligated product was used to transform *ECOS* competent *E. coli* JM109 cells (Nippon Gene, Tokyo, Japan) according to the manufacturer’s instructions. We randomly selected 63 clones from ^13^C-labeled samples and 92 clones from unlabeled samples. The 16S rRNA segments were sequenced on a 3130xl Genetic Analyzer (Applied Biosystems) using the BigDye Terminator V3.1 cycle sequencing kit (Applied Biosystems). The 16S rRNA gene sequences obtained was compared with those in the nucleotide sequence database by using the BLAST program^1^. Chimeric structures were detected by separately analyzing the phylogenies of terminal stretches at the 5′ and 3′ ends, known as fractional treeing (Ludwig et al., 1998).

### Analysis of Bacterial Communities in Pig Manure Slurry Samples using Pyrosequencing

#### Preparation of Aerated Pig Manure Slurry from Different Pig Farms

To analyze bacterial communities in aerated pig manure slurry, samples from four different locations (designated as R, N, O, and K) were collected. N corresponded to a pig fattening house at the National Institute of Livestock and Grassland Science, and R, O, and K were pig farms located in various areas of Japan separated by a distance of at least 300 km. Pig feces were transported to Hokkaido Agricultural Research Center in a cold box and slurry samples were prepared as described above. Batch aerobic treatment of the slurry was also performed as described, except that a 3-l volume was used. To prevent bacterial cross-contamination between samples, the jar fermenter including sensors and air inlet were autoclaved prior to each batch aerobic treatment. Air was supplied through a filter with a pore size of 0.22 μm.

#### Barcoded Pyrosequencing of Bacterial 16S rRNA Genes

Total genomic DNA from pig manure slurry was extracted using the UltraClean Fecal DNA Isolation kit (MO BIO, Carlsbad, CA, USA) according to the manufacturer’s instructions. The V4–V5 regions of the bacterial 16S rRNA gene were amplified using primers T6m563F (5′-NNNNNN-AYTGGGYDTAAAGNG-3′) and T6m926R (5′-NNNNNN-CCGTCAATTCMTTTRAGT-3′), where NNNNNN denotes a unique 6-mer barcode. PCR amplification was performed on a T100 thermal cycler (Bio-Rad, Hercules, CA, USA) in a 50-μl reaction volume containing the BIOTAQ HS DNA Polymerase mixture (Bioline, London, UK), 1 μl genomic DNA, and 20 pmol of each primer. The reaction conditions were as follows: 95°C for 10 min; 20 cycles of 94°C for 30 s, 52°C for 30 s, and 72°C for 1 min; and 72°C for 5 min. PCR products were resolved by electrophoresis on a 1.5% agarose gel and the DNA band of the correct size was excised and purified using the MonoFas DNA purification kit. Equal amounts of purified PCR product were pooled and ligated with adapters using the GS Titanium Rapid Library Preparation kit (Roche, Mannheim, Germany). Pyrosequencing was carried out on a 454 GS Junior instrument (Roche) following the Roche Amplicon Lib-L protocol, and the resulting sequencing reads were analyzed using the Ribosomal Database Project (RDP) pyrosequencing pipeline^2^ (Wang et al., 2007) with default parameters (maximum number of Ns = 0 and minimum average quality score = 20). Pyrosequencing reads were assigned to specific samples based on their unique bar codes; these as well as primers were then trimmed off. DECIPHER was used to identify chimeric sequences^3^ (Wright et al., 2012). Taxonomic assignment of bacterial high quality reads was performed using the RDP naive Bayesian rRNA Classifier with 80% confidence thresholds.

### Phylogenetic Analyses and Deposition of Identified 16S rRNA Genes

#### Phylogenetic Analyses of 16S rRNA Gene Sequences

Phylogenetic analyses were carried out using MEGA software version 6 (Tamura et al., 2013). Phylogenetic trees with reference 16S rRNA sequences (>1,400 nucleotides) and representative partial 16S rRNA sequences (i.e., ∼850 bp from clone analysis or 325 bp from pyrosequencing) obtained in this study were analyzed using the neighbor-joining method. Bootstrap values were obtained from 1,000 replicates.

#### Nucleotide Sequence Accession Numbers

Nucleotide sequence data obtained in this study have been deposited in the DNA Data Bank of Japan^4^ under accession numbers LC055721–LC055728.

## Results

### Aerated Slurry Conditions and Addition of Labeled Biomass

**FIGURE 1:**
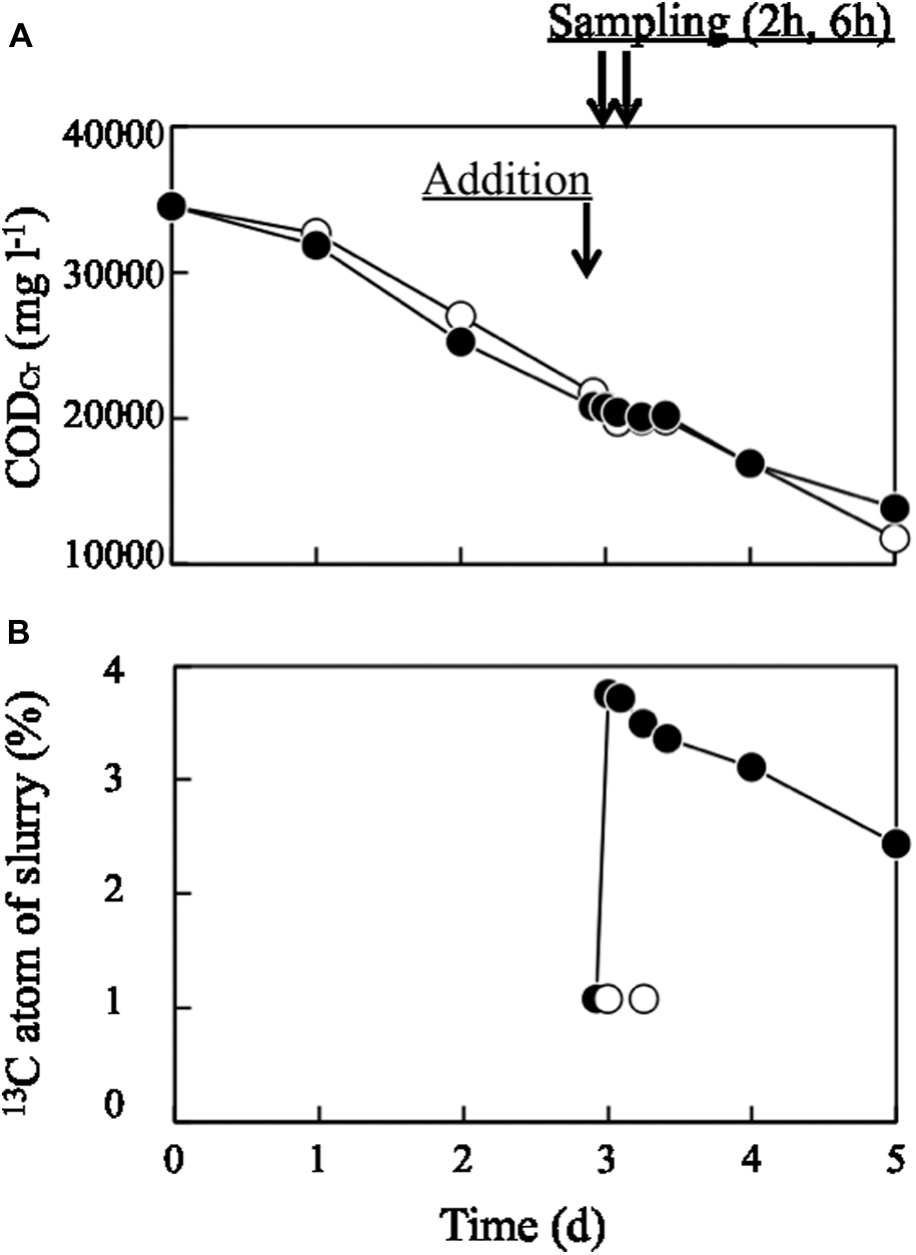
**Changes in (A) COD_Cr_ and (B) ^13^C atom% in aerated pig manure slurry.** Closed and open circles represent treatments with ^13^C-labeled and unlabeled (control) dead *Escherichia coli* cells, respectively. Arrows represent the time at which dead *E. coli* cells were added and sampling times (2 and 6 h later).

Organic matter in pig manure slurry decreased linearly from 35,000 to 15,000 mg l^-1^ over 5 days of aeration, as evaluated by COD_Cr_ (**Figure 1A**). ^13^C-labeled or unlabeled dead *E. coli* cell suspension was added on day 3; at this point, the COD_Cr_ value was approximately 21,000 mg l^-1^, while the value for ^13^C-labeled or unlabeled dead *E. coli* cell suspension was 34,510 mg l^-1^. Hence, the bacterial suspension (30 ml) corresponded to 3.3% of the total COD_Cr_ of the bulk slurry. Immediately after adding ^13^C-labeled bacterial biomass, the COD_Cr_ value of the slurry did not change significantly, while ^13^C atom% increased from 1.07 to 3.76% (**Figure 1B**) and then decreased to 2.44% on day 5. The ORP decreased to less than -400 mV within 12 h of the operation (Supplementary Figure S1A). This low ORP value lasted through day 3. After day 3, ORP began to increase and finally reached -30 mV on day 5. The pH fluctuated between 8.1 and 8.6 during the process (Supplementary Figure S1B). The time courses of ORP and pH were similar between runs incubated with ^13^C-labeled and unlabeled dead *E. coli* cells and their components.

### Bacterial Community Structures in RNA Density Gradients

**FIGURE 2:**
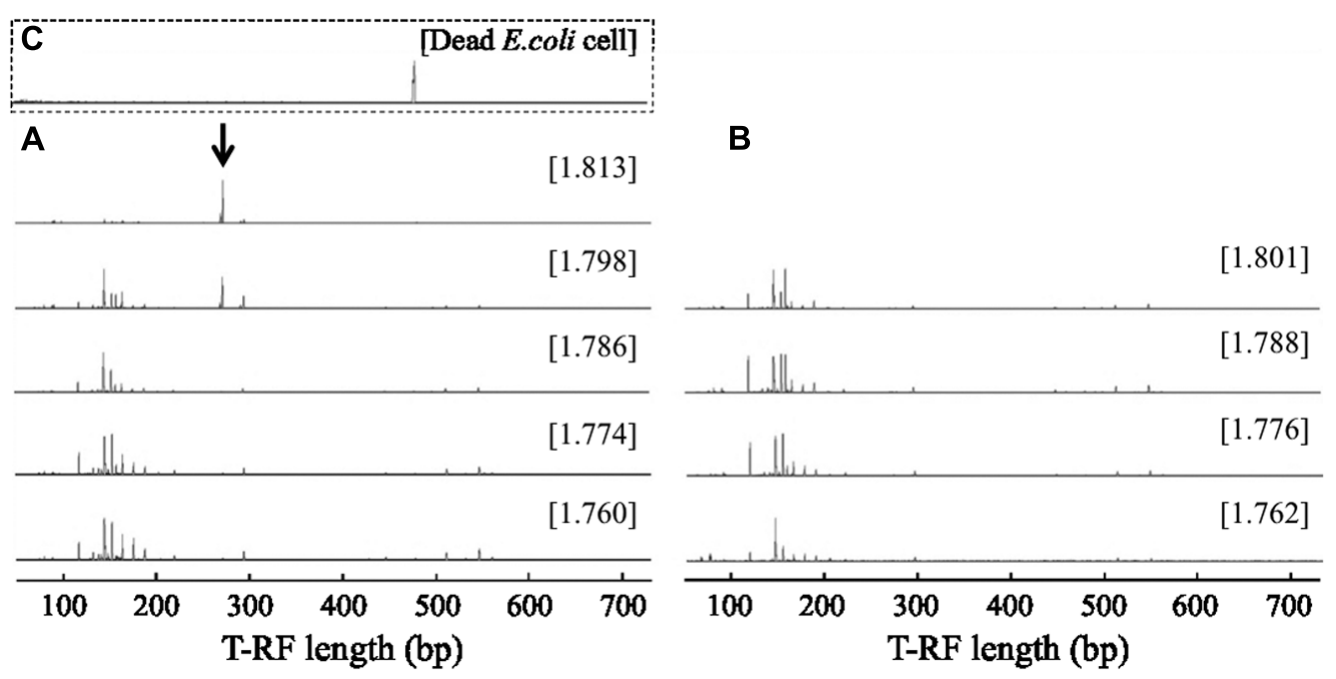
**Terminal restriction fragment length polymorphism (T-RFLP) fingerprints of bacterial 16S rRNA separated by isopycnic centrifugation from (A) ^13^C-labeled and (B) unlabeled samples 2 h after addition of dead *E. coli* cell suspension.** T-RFLP fingerprint of **(C)** the dead *E. coli* cell suspension is shown in the square with broken lines. The CsTFA BD (g ml^-1^) of each fraction is shown in square brackets. The arrow indicates specific T-RFs observed in the heavy fraction of a ^13^C-labeled sample.

RNA-based SIP was performed to identify bacterial populations capable of assimilating bacterial biomass in aerated pig manure slurry 2 and 6 h after adding dead *E. coli* cell suspension. Bacteria-specific amplicons were obtained from density fractions with the highest BD (>1.811 g ml^-1^) obtained for the ^13^C-labeled sample, while no amplicons were obtained in this fraction (>1.811 g ml^-1^) in unlabeled control samples. T-RFLP fingerprinting patterns were similar in light fractions of RNA (BD: 1.760–1.786 g ml^-1^) after a 2-h incubation (**Figure 2**). The community profiles changed with increasing BD in ^13^C-labeled samples, in which two major T-RFs (277 and 278 bp in length) predominated in heavy fractions (BD: >1.811 g ml^-1^), accounting for ∼71% of total peak heights. However, this predominance was not observed either in light fractions (BD < 1.786 g ml^-1^) of ^13^C-labeled samples or in entire-range fractions of unlabeled samples. In addition, the two T-RF peaks differed from that of the 16S rRNA from ^13^C-labeled dead *E. coli* cells (**Figure 2**). Although several minor T-RFs (94, 96, 151, 296, and 300 bp) were detected in heavy fractions, most of these were also present in light fractions. A high relative abundance of the two major peaks, reaching ∼62% of total peak heights, was still detected in heavy fractions after 6 h (Supplementary Figure S2).

### Phylogenetic Identification of Bacteria Incorporating ^13^C-labeled Bacterial Biomass

**FIGURE 3:**
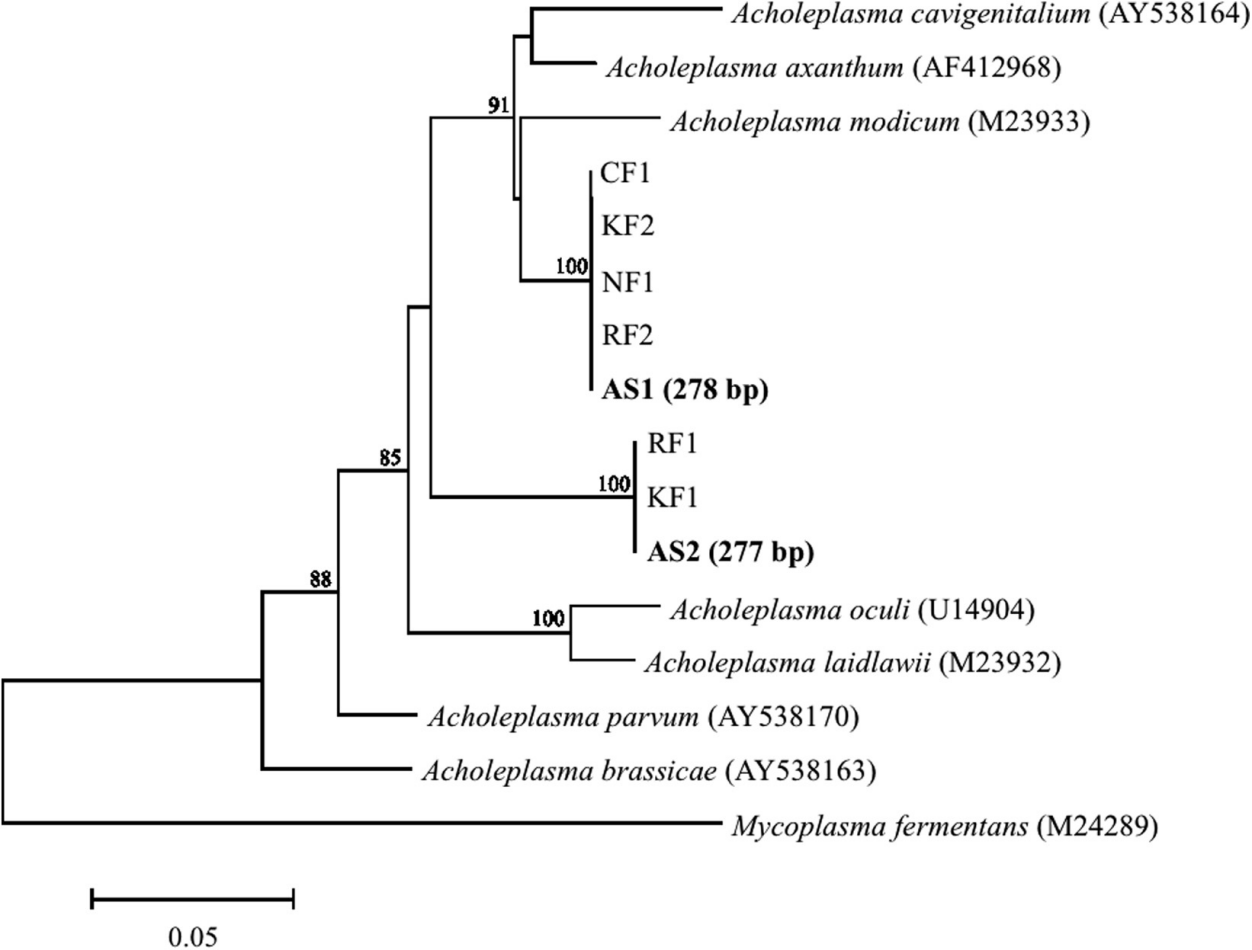
**Phylogenetic tree generated with the neighbor-joining method showing relationships among clones obtained from aerated pig manure slurry samples.** Clones were designated AS (heavy fraction of ^13^C-labeled sample, in boldface) and RF, NF, CF, or KF (sequences obtained from slurry from four different pig farms). Numbers in parentheses for AS1 and AS2 represent expected T-RF lengths of clones. Bootstrap values are shown at each node that had >70% support in a bootstrap analysis of 1,000 replicates. Scale bar represents 5% sequence divergence. DNA Data Bank of Japan/European Molecular Biology Laboratory/GenBank accession numbers of reference sequences are given.

Major populations found in heavy fractions by T-RFLP were identified by cloning and sequencing their 16S rRNAs from ^13^C-labeled and unlabeled samples. Phylogenetic affiliation and number of 16S rRNA clones are shown in **Table 1**. A high relative abundance of *Acholeplasma* sequences was present in heavy fractions of ^13^C-labeled sample as compared to unlabeled sample (63% of total clones). T-RF sizes of the sequences were estimated to be 277 and 278 bp, which corresponded to the major peaks in the T-RFLP fingerprint (**Figure 2**). These clones were related to *Acholeplasma axanthum* (90–94% sequence identity, 850 bp in length); two representative sequences, AS1 and AS2, were obtained (**Figure 3**) that belonged to clades that were distinct from the only free-living *Acholeplasma*, *A. laidlawii* (∼88% sequence identity, 850 bp in length). Besides *Acholeplasma*, specific sequences of Clostridia or Bacteroidia were obtained from the heavy fraction in ^13^C-labeled samples. However, some of these were also detected as minor T-RFs in the unlabeled T-RFLP fingerprint. A large fraction of clones in the unlabeled sample library was related to Bacilli (57% of total clones), Clostridia (12%), γ-Proteobacteria (11%), and *Corynebacterium* (11%); only one clone related to *Acholeplasma* was obtained.

**Table 1:** Phylogenic affiliations, T-RF length, and number of 16S rRNA clones obtained from the highest-density fraction of bacterial RNA following addition of ^13^C-labeled and unlabeled bacterial biomass.

Phylogenetic group	^13^C-labeled		Unlabeled	
	Clones (n)	T-RF (bp)	Clones (*n*)	T-RF (bp)
*Acholeplasma*	39	**277, 278 (71%)**, 279	1	278
Bacilli				
*Bacillus*	3	153, 165, 168	11	155, 167, **169 (4%)**
Planococcaceae	1	151	25	145, 147, **151 (25%)**, 159, 558
*Lactobacillus*	–	–	10	29, **181 (2%)**
*Streptcoccus*	–	–	6	**555 (3%)**
Clostridia				
*Sporanaerobacter*	4	296	–	–
*Tissierella*	2	300	–	–
*Proteiniborus*	1	167	–	–
*Clostridium* XI	–	–	6	**195 (5%)**
*Clostridium sensu stricto*	–	–	2	520
*Cryptanaerobacter*	–	–	2	508
*Clostridium* XlVa	–	–	1	287
Bacteroidia				
*Petrimonas*	6	**94 (3%)**	–	–
*Proteiniphilum*	2	96	1	96
α-Proteobacteria	2	150	2	439
β-Proteobacteria	–	–	4	129, 488, 496
γ-Proteobacteria	1	123	10	**123 (10%)**, 226
*Erysipelothrix*	1	173	—	—
*Corynebacterium*	–	–	10	**165 (25%)**
*Flavobacterium*	–	–	1	29
**Total**	62		92	

*T-RF detected for more than five clones within a group is indicated in boldface. Relative abundance of corresponding peaks in the T-RFLP profile is presented in parentheses.*

### Prevalence of *Acholeplasma* in Different Sources of Aerated Pig Manure Slurry

To evaluate the prevalence of *Acholeplasma* bacteria, pig manure slurry was obtained from four different locations (R, N, O, and K) in Japan. The pig manure slurry was sampled 3 days after the start of aeration; the sampling time was same as for SIP. Bacterial community composition was analyzed by pyrosequencing of 16S rRNA genes. The relative abundances of the *Acholeplasma* sequences (323 or 325 bp in length) in the four sample libraries were as follows: R, 4.5% (29 out of 649 sequences); N, 0.6% (10 out of 1583 sequences); O, 0.2% (3 out of 1431 sequences); and K, 3.9% (44 out of 1128 sequences). All representative sequences obtained from these libraries were similar to the bacteria (i.e., AS1 or AS2) obtained in the ^13^C-labeled sample (100% sequence identity, 323 or 325 bp in length) (**Figure 3**).

## Discussion

Forced aeration of pig manure slurry has been used to mitigate odor emission and stabilize slurry quality. During this process, the bacterial community—mainly comprising pig gastrointestinal bacteria in raw manure—immediately shifts to become predominantly composed of Bacilli (Hanajima et al., 2009). *Bacillus* species are ubiquitous and are typically but variably capable of growing aerobically on complex, low molecular mass substrates (Sneath, 1986). These bacteria are implicated in organic matter degradation under aeration, which converts a portion of decayed bacterial biomass to carbon dioxide, ammonia, and/or water; however, it was assumed that a significant fraction of decayed bacterial biomass is recycled by newly proliferating populations. If the slurry is mixed thoroughly under stirring and forced aeration within the reactor, most microorganisms have access to bacterial cell components (e.g., cell wall and membrane, as well as intracellular materials) from decayed bacterial cells. However, with longer incubation times, carbon derived from labeled microbial products rapidly spread throughout the slurry microorganisms via trophic interactions. We therefore focused on identifying the first-to-arrive scavengers in the aerated slurry, and demonstrated by SIP of rRNA that dead cells and their components were incorporated specifically by *Acholeplasma*.

Dead cells and their components were added just prior to ORP elevation. ORP is closely linked to the level of dissolved oxygen (DO) in slurry, and a decline in ORP has been attributed to increased DO consumption by microorganisms (Hanajima et al., 2009). In contrast, a reduction in available carbon in slurry decreases the requirement for oxygen in the decomposition of carbon substrates. This fluctuation in ORP is generally observed during aerated slurry treatment, and drastic shifts in bacterial community composition occur along with changes in ORP (Hanajima et al., 2009, 2011). Under these conditions, a certain amount of dead bacterial cells is likely produced in response to drastic shifts in the bacterial community. To prevent the continued existence and/or proliferation of ^13^C-labeled *E. coli* in aerated slurry, cell cultures were inactivated before their addition as substrate. Given that the bacterial suspension was <3.3% of the total COD_Cr_ of bulk slurry, it had little influence on the aerobic treatment process. Moreover, *E. coli* suspensions were not contaminated with *Acholeplasma* and none of the T-RFs derived from ^13^C-labeled *E. coli* were detected in the T-RFLP fingerprint. Therefore, sequences enriched in heavy fractions of RNA were derived from metabolically active bacteria that assimilated the ^13^C-labeled dead cells and their components. It was noted that these heavy fractions contained *Acholeplasma* sequences. Because none of the *Acholeplasma*-related T-RFs were observed in the unlabeled control or light fractions of the ^13^C-labeled sample, it was concluded that *Acholeplasma* was directly involved in the utilization of the dead cells and their components, albeit at a small population size.

Bacteria of the class *Mollicutes*, which includes the genus *Acholeplasma*, are widespread in nature and engage in a parasitic lifestyle; they are characterized by the absence of a cell wall and drastic reduction of genome size (Razin et al., 1998). *Acholeplasma* spp. possessing relatively large genomes of 1.5–1.8 Mbp (Lazarev et al., 2011), do not require sterols for cultivation, and are able to synthesize fatty acids from precursors, unlike other mycoplasma (Saito et al., 1977). *Acholeplasma* members have been detected as parasitic bacteria in nearly all organisms, including plants, crustaceans, mammals, and insects (Atobe et al., 1983; Clark et al., 1986; Tully et al., 1994; Chen et al., 2011). One of the best-characterized species is *A. laidlawii*, which is regarded as a universalist that adapts to various environmental conditions. It was isolated from wastewater and is the only known *Acholeplasma* capable of surviving and reproducing in animals and plants through a parasitic lifestyle and in wastewaters through a free-living lifestyle (Lazarev et al., 2011). However, *Acholeplasma* sequences obtained in this study constituted a clade distinct from that of *A. laidlawii*, demonstrating for the first time that another free-living *Acholeplasma* species exists and is metabolically active in aerated slurry. *Acholeplasma* sequences have been previously detected in swine waste lagoons (Goh et al., 2009), aerated pig manure slurry (Hanajima et al., 2009), and pigpen pit slurry (Hwang et al., 2014). The latter study found a relative abundance of 3.5% from *Acholeplasma* in pig manure slurry, which is in accordance with results from our survey of four different farm samples (∼4.4%). These results strongly suggest that *Acholeplasma* is prevalent in the pig manure slurry environment. In addition to these environments, *Acholeplasma* were found in marine sediments (Masui et al., 2008; Imachi et al., 2011), suggesting that these organisms are concentrated in copiotrophic environments in which bacterial cell components released from decayed microbial biomass accumulate.

It is widely known that Mollicutes have limited biosynthetic capabilities (Fraser et al., 1995; Sasaki et al., 2002; Vasconcelos, 2005); these are primarily for energy acquisition, with synthetic pathways being considerably reduced or absent. As such, many nutrients must be obtained from external sources (e.g., the host organism). Metabolic and genomic analyses have been carried out to elucidate the principles of metabolic regulation and adaptation to environmental conditions by Mollicutes (Lazarev et al., 2011; Kube et al., 2014; Vanyushkina et al., 2014). The *Acholeplasma* genome encodes a wide variety of ATP-binding cassette (ABC) transporters, and most genetic modules are implicated in the conversion of intermediates and are linked to ABC transporter genes for the uptake of oligopeptides and amino acids (Kube et al., 2014). This demonstrates that *Acholeplasma* are capable of incorporating oligopeptides and amino acids released from dead cells and their components. However, this raises questions about the assimilation of labile carbon sources by heterotrophic bacteria in slurry, such as why only the rRNA recovered from *Acholeplasma* was ^13^C-labeled despite most heterotrophic bacteria having access to dead cells and their components, and why ^13^C-labeled rRNA was recovered after a very short (2-h) incubation period. *Acholeplasma* has the capacity to generate dNTPs from adenosine, guanosine, uracil, and thymine (Kube et al., 2014), and *A. laidlawii* was reported to incorporate nucleic acid precursors during the first 4 h of incubation in medium (McIvor and Kenny, 1978). The detection of ^13^C-labeled rRNA within a 2-h incubation period leads us to speculate that *Acholeplasma* incorporated ^13^C-labeled nucleic acid precursors released by dead *E. coli* cells. Although most heterotrophic bacteria may incorporate ^13^C-labeled dead cells and their components, a longer incubation time would be required to obtain ^13^C-labeled rRNA from these bacteria, with the exception of *Acholeplasma*.

*Acholeplasma axanthum*, the closest relative to metabolically active bacteria identified in our study, has been isolated from a variety of habitats and hosts, including the lung of swine (Stipkovits et al., 1974). The genotypic patterns of these strains differed markedly from one another, suggesting considerable heterogeneity within the same species (Razin et al., 1983). Growth in varied environments requires regulatory switches and cross-talk between metabolic pathways to allow rapid adaptation to environmental changes in nutritional flux. The *A. laidlawii* genome contains genes encoding components of signal transduction pathways and the SOS response system, which are closely related to the regulation of gene expression and mutagenic response to stress (Lazarev et al., 2011). These functions allow *A. laidlawii* to adapt to various environmental conditions. It is unclear whether the *Acholeplasma* spp. detected in this study originated from the body of pigs or from the manure slurry itself; nonetheless, the results suggest that *Acholeplasma* has a competitive advantage for utilizing dead bacterial biomass more rapidly relative to other microbial populations.

Linking the phylogeny and function of microorganisms is one of the most fundamental challenges in microbial ecology. Generating defined mixed cultures of isolated microorganisms is one promising approach to achieve this end (Kato et al., 2005, 2014). However, in natural and engineered environments, unidentified microbial components may nonetheless play critical ecophysiological roles. In this study, we identified the first-to-arrive scavengers in aerated pig manure slurry by ^13^C-labeled bacterial biomass probing of rRNA. *Acholeplasma* spp. were identified for the first time as a major heterotroph that assimilates the dead cells and their components in pig manure slurry. Substrate competition has been found in many types of biological processes, in which physiologically similar microorganisms co-exist and fill ecological niches owing to the difference in the substrate availability and affinity (Hori et al., 2006; Aoyagi et al., 2015). Due to the limited biosynthetic capabilities, free-living *Acholeplasma* spp. most likely require biomolecules released from dead bacterial cells as substrates. Actually, they were capable of incorporating the dead cells and their components more rapidly than the other microbial populations, which allowed the survival and prevalence of *Acholeplasma* spp. in pig manure slurry. These findings provide deeper understanding of carbon flow mediated by microorganisms in copiotrophic environments and unveil one of the mechanisms underlying the microbial ecosystem development.

## Author Contributions

DH designed the research. DH and TH wrote the manuscript. DH, TA, and TH carried out the experiments and analyzed the data.

## Conflict of Interest Statement

The authors declare that the research was conducted in the absence of any commercial or financial relationships that could be construed as a potential conflict of interest.

## Footnotes

1 http://ddbj.nig.ac.jp/blast/

2 http://pyro.cme.msu.edu/

3 http://decipher.cee.wisc.edu/

4 http://www.ddbj.nig.ac.jp
