# Supplementary material for: Survival of free-living Acholeplasma in aerated pig manure slurry revealed by 13C-labeled bacterial biomass probing
Source: Front Microbiol. 2015 Oct 31;6:1206. doi: 10.3389/fmicb.2015.01206 (PMC4628116; doi:10.3389/fmicb.2015.01206)
Supplement: Supplementary file 1 [file Data_Sheet_1.PDF]

Supplementary Material

Survival of free-living *Acholeplasma* in aerated pig manure slurry revealed by ^{13}C-labeled bacterial biomass probing

Dai Hanajima[1]*, Tomo Aoyagi[2], Tomoyuki Hori[2]

[1]Dairy Research Division, Hokkaido Agricultural Research Center, National Agricultural and Food Research Organization, Sapporo, Japan
[2]Environmental Management Research Institute, National Institute of Advanced Industrial Science and Technology, Tsukuba, Japan

*** Correspondence:** Dai Hanajima, Dairy Research Division, Hokkaido Agricultural Research Center, National Agricultural and Food Research Organization, 1 Hitsujigaoka, Sapporo 062-8555, Japan.

Email:hanadi@affrc.go.jp

1. **Supplementary Figures**

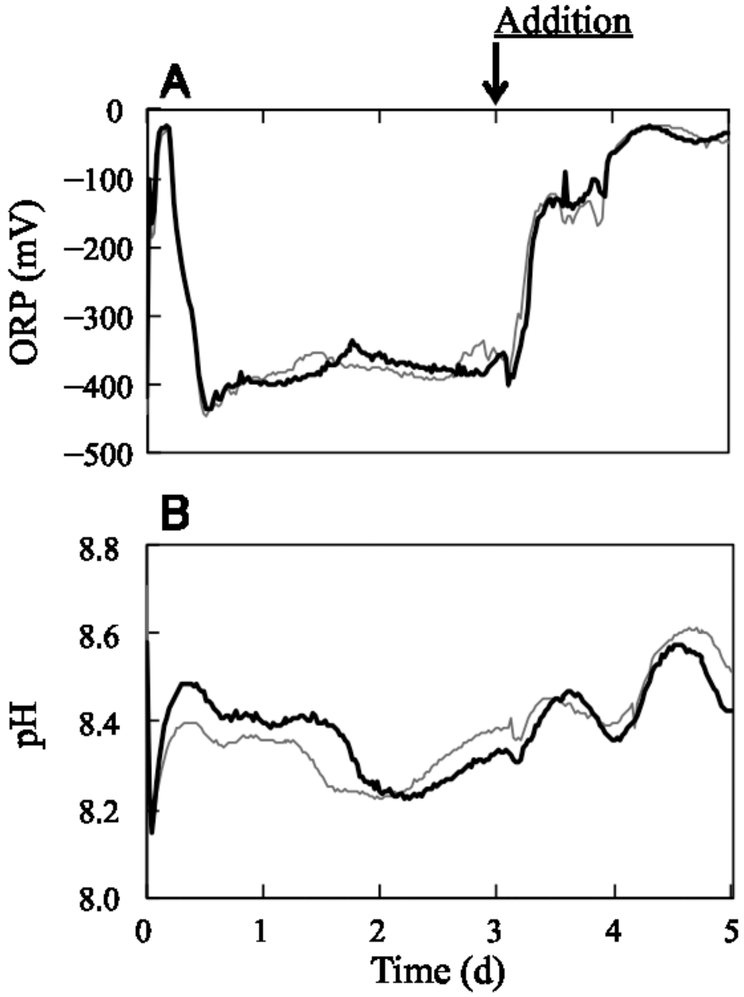

Supplementary Figure 1. Changes in (A) ORP and (B) pH in aerated pig manure slurry. Thick and thin lines represent treatments with ^{13}C-labeled and unlabeled (control) *E. coli* cells, respectively. Arrow represents the time at which decayed *E. coli* cells were added.

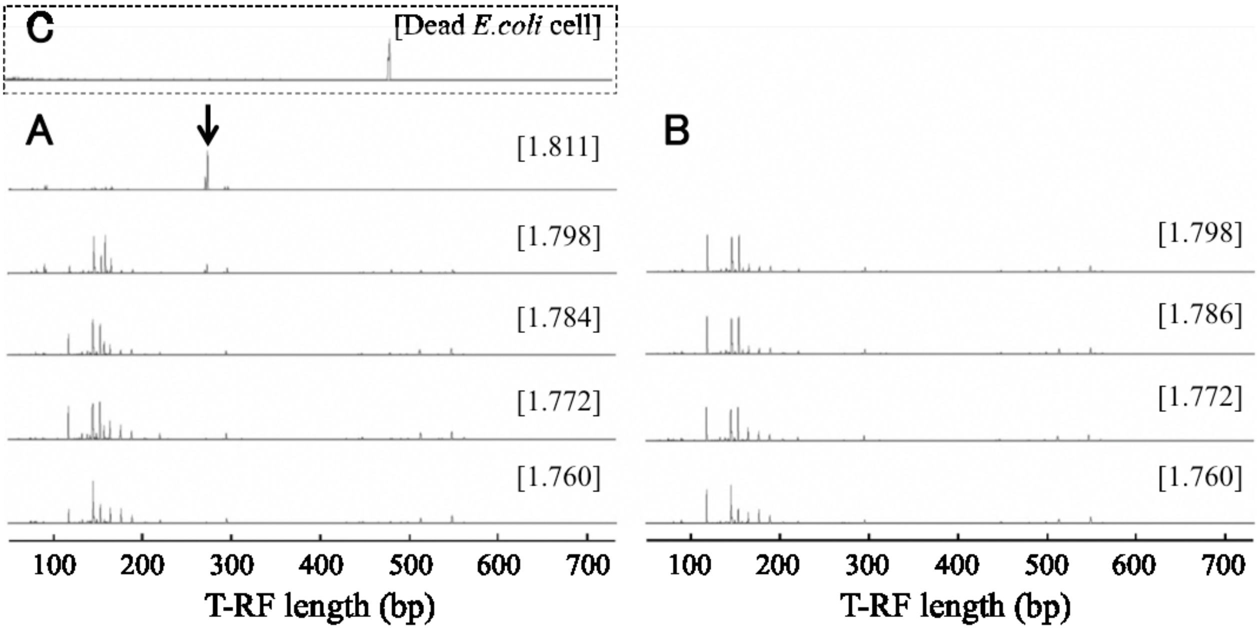

Supplementary Figure 2. T-RFLP fingerprints of bacterial 16S rRNA separated by isopycnic centrifugation from (A) ^{13}C-labeled and (B) unlabeled samples 6 h after addition of dead *E. coli* cell suspension. T-RFLP fingerprint of (C) a decayed *E. coli* cell suspension is shown in the square with broken lines. The CsTFA BD (g ml^{-1}) of each fraction is shown in square bracket. The arrow indicates specific T-RFs observed in the heavy fraction of ^{13}C-labeled sample.
